# Dynamics of a neuronal pacemaker in the weakly electric fish *Apteronotus*

**DOI:** 10.1038/s41598-020-73566-3

**Published:** 2020-10-07

**Authors:** Aaron R. Shifman, Yiren Sun, Chloé M. Benoit, John E. Lewis

**Affiliations:** 1 Department of Biology, University of Ottawa, Ottawa, Ontario K1N 6N5 Canada; 2 Center for Neural Dynamics, University of Ottawa, Ottawa, Ontario K1N 6N5 Canada; 3 uOttawa Brain and Mind Research Institute, Ottawa, Ontario K1H 8M5 Canada

**Keywords:** Biophysical models, Dynamical systems

## Abstract

The precise timing of neuronal activity is critical for normal brain function. In weakly electric fish, the medullary pacemaker network (PN) sets the timing for an oscillating electric organ discharge (EOD) used for electric sensing. This network is the most precise biological oscillator known, with sub-microsecond variation in oscillator period. The PN consists of two principle sets of neurons, pacemaker and relay cells, that are connected by gap junctions and normally fire in synchrony, one-to-one with each EOD cycle. However, the degree of gap junctional connectivity between these cells appears insufficient to provide the population averaging required for the observed temporal precision of the EOD. This has led to the hypothesis that individual cells themselves fire with high precision, but little is known about the oscillatory dynamics of these pacemaker cells. As a first step towards testing this hypothesis, we have developed a biophysical model of a pacemaker neuron action potential based on experimental recordings. We validated the model by comparing the changes in oscillatory dynamics produced by different experimental manipulations. Our results suggest that this relatively simple model can capture a large range of channel dynamics exhibited by pacemaker cells, and will thus provide a basis for future work on network synchrony and precision.

## Introduction

Timing of neuronal spikes is critical to many brain processes, including sound localization^1–3^, escape responses^4–6^, and learning and memory^7,8^. When neural processes are periodic, they can form the basis for biological clocks which span a range of precision (variability in oscillation period), with a higher variability leading to a less reliable clock. Variability in the period of neuronal oscillators (reported as a coefficient of variation: CV = s.d./mean) can be relatively high, as in the bullfrog sciatic nerve with a CV = 0.37^9,10^. For reference, a random Poisson process has a CV = 1, while cortical neurons can have a CV > 1^11,12^. In contrast, the neural oscillators underlying the electric organ discharge (EOD) of the weakly electric fish *Apteronotus* have a CV as low as $\sim 10^{-4}$ (corresponding to a standard deviation of $\sim \,$100ns), making it the most precise biological oscillator known^10,13^. The high precision of the EOD of *Apteronotus* therefore makes it a particularly attractive model for the study of neural circuit timing^10,13,14^.

*Apteronotus* generates an oscillating electric field (EOD) to sense their environment in the dark^15^. Objects in the environment interact with the EOD causing field perturbations which are sensed by electroreceptors on the skin. The timing of the oscillations underlying the EOD are set by the medullary pacemaker network (PN)^13,16–19^. This nucleus is a collection of two principle cell types: pacemaker cells, which are oscillatory cells and relay cells which project down the spinal cord to drive the EOD^14,16,20^. Additionally, there are parvalbumin positive cells (parvocells) whose function is currently unknown, but are not thought to contribute to the oscillatory function of the PN^21^.

Within the PN, the pacemakers cells are highly synchronized, with relative phases across cells close to 2% of the oscillator period^10,14^. In general, networks are thought to achieve high-precision and high-synchrony through the population-averaged activity of a large number of strongly-connected cells^14,16,22^. However, pacemaker and relay cells are connected only sparsely, with weak gap junctions^14,16–18^. Although network connectivity may be functionally enhanced through the electric feedback from the EOD itself^14,23^, the apparent disconnect between high synchrony/precision and low connectivity in the PN may also be explained by the high precision of individual cells^16^, with synchrony emerging from weak interactions between precise cells with stereotyped dynamics. Indeed, some underlying oscillatory dynamics are thought to be more amenable to synchronization than others^24–26^.

Previous studies have used a Hodgkin–Huxley based model to explore PN synchrony and precision^14,16^, but this model was not intended to accurately represent the action potential waveform of pacemaker neurons. While these studies provided insight into pacemaker network interactions, a more accurate biophysical model is required to determine how transmembrane currents, intrinsic oscillatory dynamics and gap junctional coupling impact single cell precision and network synchrony. To this end, we present a biophysically based pacemaker cell model which accurately captures the waveform of pacemaker cells as well as their dynamical responses to experimental manipulations.

## Results

### Model fit

**Figure 1:**
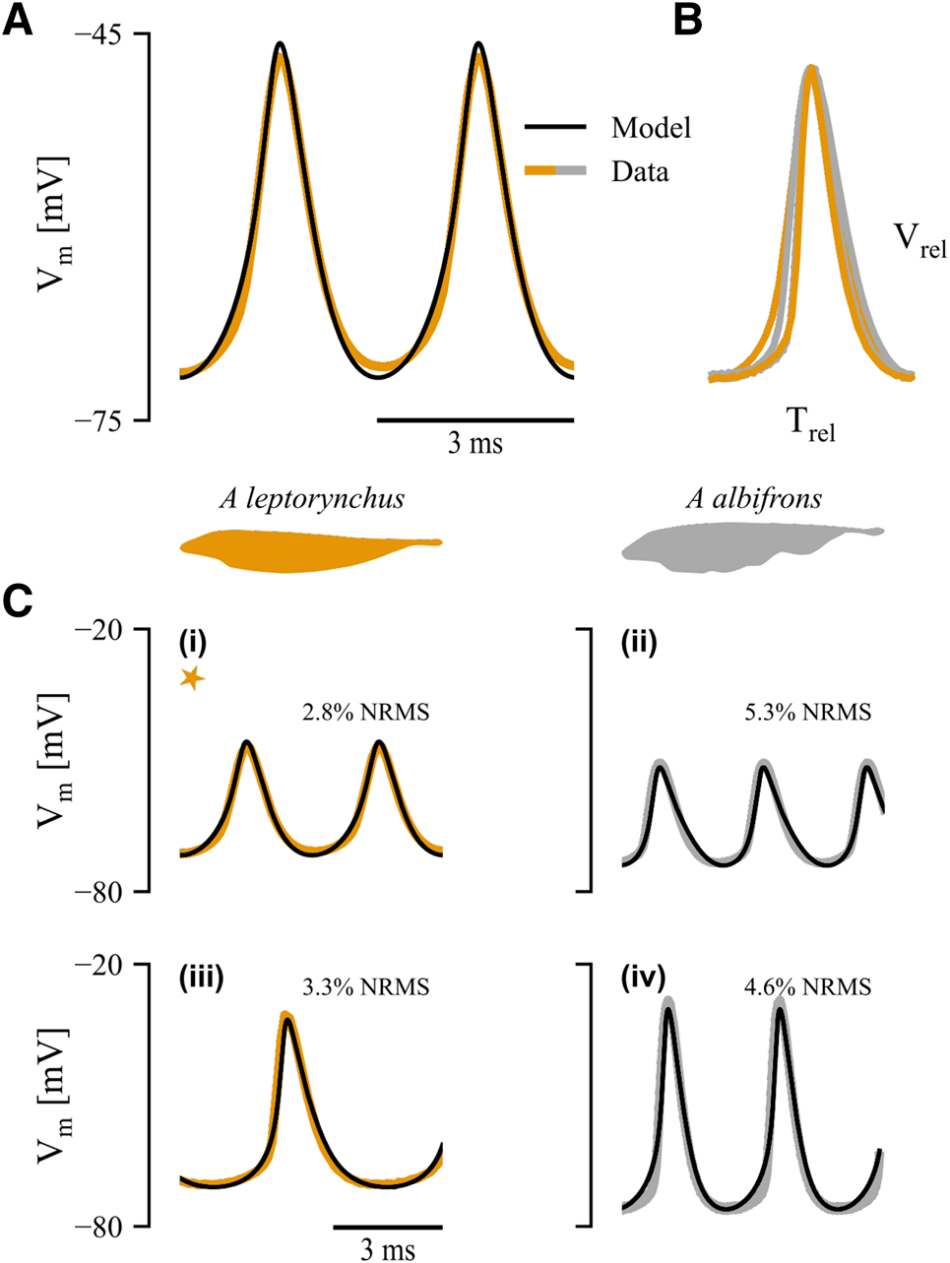
Model fit results for *A. leptorhynchus* (orange) and *A. albifrons* (grey). (**A**) Canonical model fit (black line) to A. leptorhynchus action potential waveform (orange). (**B**) Dimensionless waveform (action potential normalized by period in time and peak–peak amplitude) from two individuals from each species. (**C**) Data fits showing model flexibility over a range of frequencies, amplitudes and means for *A. leptorhynchus* (left) and *A. albifrons* (right). Orange star indicates model fit in panel A.

We developed a biophysical Hodgkin–Huxley-based model of a pacemaker neuron in the PN of a weakly electric fish. Motivated by previous studies^27^, our model included voltage-dependent sodium, potassium, and calcium channels, along with leak channels ($\hbox {I}_{\mathrm{Na}}$, $\mathrm{I}_{\mathrm{K}}$, $\hbox {I}_{\mathrm{Ca}}$, $\mathrm{I}_{\mathrm{L}}$). We used a differential evolutionary algorithm (DEA) to survey a 44-dimensional parameter space (see Methods and Appendix). After optimization, the RMS error between model and data waveform was 0.7 mV; when normalized by action potential amplitude, this corresponds to a 2.8% error. Note that small differences in action potential timing can lead to relatively large errors due to the fast rise and fall times that are typical of action potentials, so the model matches the data even better than the RMS error would suggest over most of the action potential cycle (Fig. 1A). For model fits see Tables S2 and S3.

After the initial fitting process, our model described an action potential of a single cell from an individual *A. leptorhynchus*. It is also of interest to determine how well this model will generalize across individuals and to the related species *A. albifrons*. In Fig. 1B, we show action potential waveforms (dimensionless, normalized in both time and amplitude) from pacemaker cells from two individuals of each species (Fig. 1B); the similarity across waveforms suggests that the underlying dynamics are also similar. To demonstrate this and to show the flexibility of the model, we refit the model to each of these four action potential waveforms with the same parameter bounds (Fig. 1C; data from Fig. 1A is indicated by gold star). Over a range of amplitudes and frequencies, the model fits involved a worst-case error of 5.3%. And importantly, there were no systematic differences in the voltage dependence of the gating variables across all models (Supplemental Figure S1).

**Figure 2:**
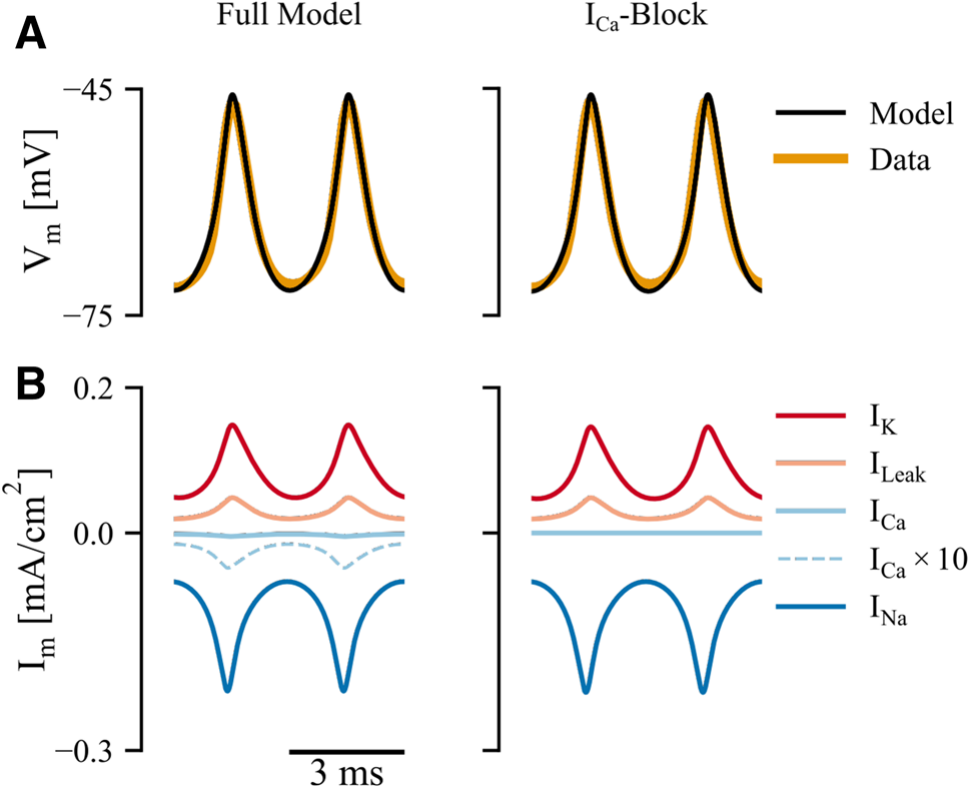
Analysis of currents in canonical model (Fig. 1A) and in model with $\hbox {I}_{\mathrm{Ca}}$-block. (**A**) Model fits for both full model (left) and blocked model (right) showing no systematic differences. (**B**) Current breakdown with $\hbox {I}_{\mathrm{Ca}}\times$10 (dashed light blue line) showing a 10 $\times$ magnified calcium current for illustrative purposes.

Previous results suggest that while calcium can contribute to action potential waveform shape, it does not fundamentally underlie pacemaker cell oscillation^27^. This is consistent with the relatively low magnitude of $\mathrm{G}_{\mathrm{Ca}}$ in our models. We tested this further by setting $\hbox {G}_{\mathrm{Ca}}$ to 0 ($\hbox {I}_{\mathrm{Ca}}$-Block ) and found that the model continues to oscillate with only subtle changes to the waveform (Fig. 2A). In Fig. 2B, we show the contributions of each current to the action potential waveform. As expected, the depolarization of the action potential is driven by the sodium current, the repolarization/hyperpolarization is driven by potassium current, and as just mentioned, $\hbox {I}_{\mathrm{Ca}}$ has little effect (compare the full model with the $\hbox {I}_{\mathrm{Ca}}$-Block model, Fig. 2B). Overall, our modeling results confirm previous experimental results suggesting a minimal role of calcium in the pacemaker action potential oscillation.

Models having a large number of parameters are at risk of being overfit, and since we have used a biophysical model with 44 parameters to fit a single oscillator waveform, a good fit would not be surprising. Therefore, it is important to validate the dynamics of our model against additional data. To this end, we consider the pacemaker oscillatory dynamics during two different experimental manipulations: low concentration of extracellular sodium (decreased $\mathrm{E}_{\mathrm{Na}}$), and pharmacological block of Na$^+$ and K$^+$ channels^27^.

### Model validation: effects of $\mathrm{E}_{\mathrm{Na}}$

To test the new pacemaker model, we compared its oscillatory dynamics under conditions that differed from those in the model fitting process. It is common for dynamical systems to undergo qualitative changes in behavior as a result of small changes in a system parameter. This property is referred to as a bifurcation^28^. One particular example of a bifurcation relevant to pacemaker dynamics is the transition between an oscillating state and a rest state (non-oscillating), and vice-versa; the nature of this transition depends on the system properties as well as the particular parameter that is varied.

One classic way in which oscillations can arise is through a Hopf bifurcation. The hallmark of a Hopf bifurcation is that the transition from an oscillating state to rest (or vice-versa) involves a discontinuous jump (i.e. as a system parameter is varied, there is an abrupt change in oscillation frequency from some minimum value to zero)^28^. There are two kinds of Hopf bifurcations: one that exhibits a hysteresis and one that does not, referred to as *subcritical* and *supercritical* respectively. In a *subcritical* Hopf bifurcation, hysteresis appears as a bistable system i.e. at a given parameter value the system can be oscillatory or not. Conversely in a *supercritical* case hysteresis is not present. An alternative type of bifurcation (the homoclinic bifurcation) involves a continuous transition from rest to oscillating state along with a gradual change in oscillation frequency (i.e. rest can be thought of as an oscillation with infinite period)^28^. In summary, characterizing the transition between oscillating and non-oscillating states can provide a test of system dynamics^29^.

Lowering the equilibrium potential of sodium ($\mathrm{E}_{\mathrm{Na}}$) via changes in extracellular Na$^+$ concentration typically leads to cessation of action potential generation. We thus used this parameter to explore the transition between oscillating and non-oscillating states in both our pacemaker model and in experimental pacemaker preparations. In our model, we can manipulate $\mathrm{E}_{\mathrm{Na}}$ directly. In our experiments, low-Na$^+$ ACSF was washed in to dilute the control ACSF, thereby gradually decreasing $\mathrm{E}_{\mathrm{Na}}$. Under ideal mixing conditions, the sodium concentration of the bath should obey the exponential diffusion equation (1), where *r* is the flow rate and $[\text {Na}^+_\text {out}]_0$ is the initial concentration of extracellular Na$^+$

1 $$\begin{aligned} {[}\text {Na}^+_\text {out}](t) = [\text {Na}^+_\text {out}]_0e^{-\frac{t}{r}} \end{aligned}$$

$\hbox {E}_{\mathrm{Na}}$ is given by the Nernst equation; therefore by substitution we have in Eq. (2)

2 $$\begin{aligned} E_\text {Na}\propto \log \frac{[\text {Na}_\text {out}^+](t)}{[\text {Na}_\text {in}^+ ] }=-k\frac{t}{r}+\alpha \end{aligned}$$

where $\propto$ represents proportionality, and *k* and $\alpha$ are lumped constants. This implies that $\mathrm{E}_{\mathrm{Na}}$ should decrease linearly in time, and since we do not have a direct measure of $\mathrm{E}_{\mathrm{Na}}$, time should be a good proxy.

**Figure 3:**
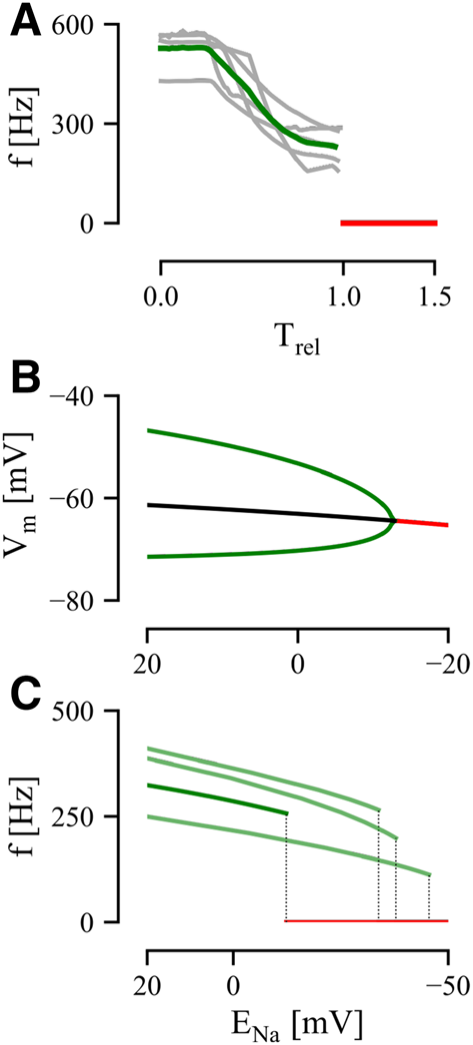
Data and model bifurcation analysis. (**A**) Relative time-series of pacemaker frequency as Na-free ACSF is washed in (see Methods) for 5 different pacemaker preparations. Green trace represents average (individual preparations in gray) and red trace represents cessation of firing. T = 1 represents bifurcation point. (**B**) Orbit diagram for model bifurcation analysis with respect to $\mathrm{E}_{\mathrm{Na}}$. Green trace is action potential extrema. Black trace is unstable fixed point and red trace is stable fixed point. Black-Red intersection point is the Hopf bifurcation. (**C**) Frequency analysis of the model Hopf bifurcation. Dark green line represents model in Fig. 1C (i). Light green lines represent results from other model fits, Fig. 1C (ii–iv). Red line represents cessation and dotted lines show the bifurcation of each model.

In Fig. 3, we show the transition between oscillation and rest in both model and experiments. Our experimental analysis reveals that pacemaker frequency decreases over time, with an abrupt shift to the rest state (for simplicity we define rest to have zero frequency), as $\mathrm{E}_{\mathrm{Na}}$ decreases (Fig. 3A). To account for variability between experiments, we normalize the time scale such that the PN ceases to oscillate at time t = 1. Measurements from individual preparations are shown in grey, with the mean shown in green (N = 5 fish). On average, we see that the oscillation stops at $\sim \,$260 Hz (Fig. 3B).

For the pacemaker model, we compute a bifurcation diagram showing the system’s state (membrane potential, Vm) for different values of $\mathrm{E}_{\mathrm{Na}}$ using XPP^30^ (Fig. 3B). As $\mathrm{E}_{\mathrm{Na}}$ decreases, the model neuron transitions from a oscillating (membrane potential extrema in green) to rest (red) at a bifurcation point corresponding to $\mathrm{E}_{\mathrm{Na}} = -12.8$ mV with no hysteresis. To distinguish a Hopf from a homoclinic bifurcation, we measured the frequency of the oscillation as $\mathrm{E}_{\mathrm{Na}}$ decreases in Fig. 3C (dark green trace). The oscillation frequency follows a square-root-like curve until a discontinuity at $\mathrm{E}_{\mathrm{Na}}=-12.8$mV, after which the cell stops firing: characteristic of a Hopf bifurcation^28^. In other words, we can say that as $\mathrm{E}_{\mathrm{Na}}$ is increased, our model undergoes a supercritical Hopf bifurcation at $\mathrm{E}_{\mathrm{Na}}=-12.8$  mV. We observe qualitatively similar dynamics for the other model fits (Fig. 1C, light green traces).

While both the models and recordings undergo a sudden loss of spiking, a feature consistent with a Hopf bifurcation, other features of the model dynamics do not match the data. First, the manner in which frequency decreases with decreasing $\mathrm{E}_{\mathrm{Na}}$ is very different, as indicated by an increasing versus decreasing second derivative (compare Fig. 3A,C). This is possibly due to nonlinear changes in $\mathrm{E}_{\mathrm{Na}}$ in the experiments resulting from variations in mixing and diffusion through the tissue; this would affect the detailed time-course of $\mathrm{E}_{\mathrm{Na}}$ but the relation between $\mathrm{E}_{\mathrm{Na}}$ and time will still be monotone. Secondly, we note that the experimental data spans a larger frequency range ($\sim \,$525 Hz$\rightarrow \sim \,$230 Hz; a drop of 60%) whereas the model spans 380–260 Hz, a drop of  30% (although for the other model fits we see 40–50% drops). This could be due to several factors, including recruitment of additional currents, or homeostatic control of the local extracellular medium. It is important to note however, that despite these quantitative differences, both model and experimental systems show similar dynamics, with each exhibiting a Hopf bifurcation.

### Model validation: pharmacological manipulations

**Figure 4:**
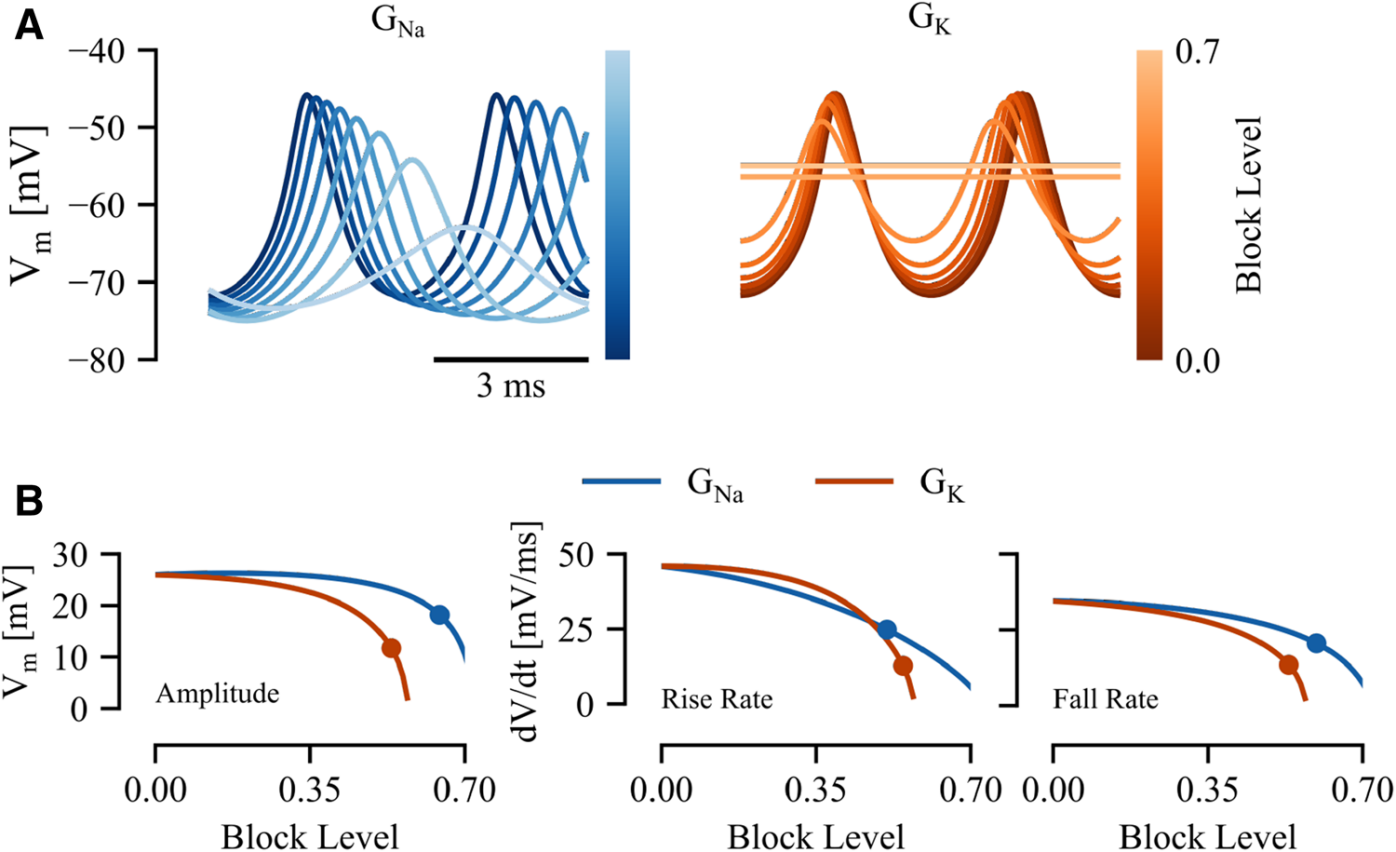
Response to progressive block of Na$^+$ and K$^+$ channels. (**A**) Model response to Na$^+$ channel block ($\mathrm{G}_{\mathrm{Na}}$, left) and K$^+$ channel block ($\mathrm{G}_{\mathrm{K}}$, right). (**B**) Action potential properties computed as a function of block level for peak-peak amplitude (left), action potential rise rate (center) and fall rate (absolute value; right). Dots represent block level with equivalent percentage change in each property from data reported in^27^.

In a similar manner, we can also consider how PN oscillatory dynamics change as individual currents are manipulated. Smith and Zakon^27^ showed that blocking either $\mathrm{I}_{\mathrm{Na}}$ or $\mathrm{I}_{\mathrm{K}}$ channels results in firing cessation in experimental preparations of *A. leptorhynchus*. Importantly, they measured action potential waveform parameters as the channel blocker was washed in (i.e. as an increasing fraction of the channels are blocked). These data can thus provide another means of model validation. We simulated this gradual channel block in the pacemaker model by manipulating the conductance of the appropriate channel as $G_\text {ion}\rightarrow (1-\beta )G_\text {ion}$ where $\beta$ is a number between 0 and 1 ($\beta =0$ represents no block, while $\beta =1$ represents complete block). We demonstrate the effects of progressive block of either sodium (left) and potassium (right) channels in Fig. 4A (block level, $\beta =0$ to $\beta =0.7$). As in the $\mathrm{E}_{\mathrm{Na}}$ experiments, we performed a bifurcation analysis in XPP^30^ and found that both of these bifurcations are supercritical Hopf bifurcations (not shown). The reader familiar with bifurcation analyses may note that these decreases in $\mathrm{G}_{\mathrm{Na}}$ appear to result in a continuous frequency drop, but over a larger parameter range the discontinuous drop in frequency associated with a Hopf bifurcation is in fact observed.

Because we do not know the equivalent block level at the time the waveform properties were measured in the experiments, we use the following qualitative comparison based on three action potential waveform features. We consider how these features vary as the block level is increased (Fig. 4B), and what block level is required to match experimental data (solid circle, Fig. 4B). A good model will be internally consistent in that the required block level should be consistent across all waveform features. In Fig. 4B, we show the peak-to-peak amplitude, peak rise rate, and peak fall rate (taken as positive for symmetry with rise rate) for both sodium and potassium block as a function of block level ($\beta$). The equivalent block level (the block level that corresponds to the percent change noted in the original data) is indicated by a solid circle. The equivalent level for sodium block is $0.59 \pm 0.06$ (mean ± standard deviation) whereas that for potassium block is $0.55 \pm 0.007$; the low variability here suggests good model performance.

Overall, our analyses show that this new model captures the main oscillatory dynamics and action potential waveforms of pacemaker cells based on the underlying sodium and potassium currents.

## Discussion

The pacemaker network (PN) of wave-type electric fish sets the timing of a neural oscillation which exhibits precision and stability far beyond that of any known biological oscillator^13,14^. To understand these dynamics, we have developed a biophysically relevant model of pacemaker neurons that reproduces the action potential waveform as well as the effects of various experimental manipulations. From a dynamical systems perspective, we show that our model undergoes a Hopf bifurcation as $\mathrm{E}_{\mathrm{Na}}$ is decreased. A similar effect is seen experimentally when Na$^+$ is removed from the extracellular medium: oscillations stop with a minimum frequency $\sim \,$260 Hz. While our data only shows the offset of spiking (and thus super and subcritical bifurcations cannot be distinguished), we find that the oscillation stops suddenly, which rules-in a Hopf bifurcation and rules out any form of homoclinic bifurcation, where the onset of an oscillation can have an arbitrarily low frequency. In addition, we were not able to successfully recover a normal oscillation after low-Na$^+$ treatment, and thus could not differentiate between sub and supercritical bifurcations based on the presence of hysteresis. This could be due to a network bistability where the control Na$^+$ level could permit both an oscillatory state and a quiescent rest state. Further work is required. Nonetheless, our model accurately reproduces the changes in waveform properties such as amplitude and peak rise rate caused by partial channel block.

Of particular interest in this study is the fact that our model was fit to data from two related species (*A. leptorhynchus* and *A. albifrons*). While there are known differences in pacemaker cell counts, and EOD frequencies^18,31–34^, little is known about the differences in pacemaker network dynamics between these species^14^. We show preliminary data to suggest that both species have an equivalent dimensionless action potential waveform (Fig. 1B) that can vary widely across amplitude, mean and frequency. The implication of this being that pacemaker cells in both species have similar dynamics. This is supported by the fact that the model was fit to these different waveforms within relatively narrow parameter bounds. Furthermore, experimental validations are done with data from both *A. albifrons* ($\mathrm{E}_{\mathrm{Na}}$) and *A. leptorhynchus* (channel block).

This model can also provide important insight into precision and synchrony in the PN. For example, fast, early currents such as $\mathrm{I}_{{\mathrm{Na}}_{\mathrm{P}}}$ tend to decrease synchrony across a network^35^, whereas some of the slow, late potassium currents^35,36^ tend to increase network synchrony^35^. Interestingly, we found that $\hbox {I}_{\mathrm{Na}_{\mathrm{P}}}$ was not required to explain pacemaker waveforms (consistent with recent molecular work^34^), and that the gating dynamics of $\mathrm{I}_{\mathrm{K}}$ converged to those similar to a delayed rectifier, with no apparent requirement for the high-threshold potassium currents often observed in high-frequency systems^34^. The specific dynamics of individual neurons in a network can also influence network synchronization^35,36^. In particular, neurons exhibiting supercritical Hopf bifurcations (also referred to as Type II excitability) can lead to more robust synchronization in some cases^25,26^. Understanding the specific roles of bifurcation structure in PN precision and synchrony will require future modeling and experimental work.

While it is important to note that the channels defined in our model were not tuned to represent the particular channel subtypes expressed in the PN^27,34^, it is nonetheless interesting to compare their dynamics. The high-threshold voltage-gated K$^+$ channel (Kv1.8) is abundantly expressed in the PN^34^. Of note, the relatively long time constant typical of these channels would seem inappropriate for the high-frequency firing of PN cells. The fast-gating potassium channels (Kv3) found in the auditory system^37^ are also present in the Apteronotid PN^34^ and appear better-suited to high frequency firing. Interestingly, both of these channel subtypes have a narrow gating window which is significantly more depolarized than the peak of the pacemaker cell waveform. While larger action potentials may be generated by relay cells or at distinct locations in the circuitous pacemaker axons, it is also possible that slower channel kinetics act to smooth out high-frequency fluctuations leading to a more “sinusoidal” current that would facilitate synchronization^38^. On the other hand, the K+ channel in our model has a relatively low activation threshold ($\sim -60$mV), but it may represent the dynamics of a suite of channels in combination, rather than any particular channel. Nonetheless, high-frequency and waveform shape can also drive adaptation^39,40^, so it is likely that the kinetics of these K+ channels show adaptations better-tuned to the high frequencies of the EOD. Indeed, the PN expresses subunits such as Kv$\beta$2 that are known to modulate channel activation time in Kv1^34^. More work on the biophysics of these channels will be required to clarify these issues.

We acknowledge that our model is a single cell model fit to data from an intact pacemaker network. The impact of this is not clear; gap junctional strength is proportional to the voltage between cells, so in a synchronized network, the voltage difference between cells is low, with gap junctional coupling serving primarily as an error-correcting entraining force. This suggests that, at least to first order, the network effects of gap junctions may be minimal. Nonetheless, given the model exhibits similar oscillatory dynamics to those observed experimentally, it will provide a basis for future work focused on how intrinsic neuronal dynamics interact with gap junctional coupling to produce high temporal precision and synchrony in the pacemaker network.

## Methods

### Model developement

Previous pharmacological experiments have suggested that pacemaker cells express the following suite of ionic currents: inactivating sodium ($\mathrm{I}_{\mathrm{Na}}$) and/or persistent sodium ($\hbox {I}_{\mathrm{Na}_{\mathrm{P}}}$), inactivating potassium ($\mathrm{I}_{\mathrm{K}}$), T/R type calcium ($\mathrm{I}_{\mathrm{Ca}}$), and leak ($\hbox {I}_{\mathrm{L}}$)^27^. Using the standard Hodgkin–Huxley-style biophysical approach^41^, these currents underlie the dynamics of our model (Eqs. 3–7), where *v* is the membrane potential, *b*, *m*, and, *n* are calcium, sodium, and potassium channel activation variables, and *g*, *h*, and, *q* are the respective inactivation variables. For full specification see supplemental materials.

3 $$\begin{aligned} \frac{dv}{dt}&= -I_{Leak} - I_{Ca} - I_{Na} - I_K \end{aligned}$$

4 $$\begin{aligned} I_{Leak}&= G_{Leak}( v-E_{Leak} ) \end{aligned}$$

5 $$\begin{aligned} I_{Ca}&= G_{Ca}b^2g^2( v-E_{Ca} ) \end{aligned}$$

6 $$\begin{aligned} I_{Na}&= G_{Na}mh( v-E_{Na} ) \end{aligned}$$

7 $$\begin{aligned} I_K&= G_Kn^2q^2( v-E_{K} ) \end{aligned}$$

Model parameters were fit to intracellular recordings from pacemaker cells in *Apteronotus albifrons* (see experimental methods) and in *Apteronotus leptorhynchus* (published previously^14^). A standard waveform from a representative pacemaker cell comprising two successive action potentials, averaged over 30 sweeps, was used to fit the primary model. Fitting two successive action potentials (oscillator cycles), rather than one, minimizes frequency drift between model and target waveforms. We also show that the primary model can be generalized by fitting it to waveforms from other pacemaker cells in both *Apteronotus leptorhynchus* and the related species *Apteronotus albifrons* (see Fig. 1). Note that Smith and Zakon (2000) also suggested a role for a persistent sodium current ($\hbox {I}_{\mathrm{Na}_{\mathrm{p}}}$), but our preliminary studies showed that including this current resulted in many solutions that would not spike (not shown), so we did not include $\hbox {I}_{\mathrm{Na}_{\mathrm{p}}}$ in our final model. Subsequent work showed that $\hbox {I}_{\mathrm{Na}_{\mathrm{p}}}$ is not strongly expressed in the PN of either Apteronotid species^34^. While low levels of expression of this current could nonetheless provide a means of modulating PN frequency, it is not necessary to explain variations in pacemaker cell waveform across individuals and species.

We implemented a generic, parameterized model in the Brian2 simulation engine (version 2.3)^42^, and fit our model using a differential evolutionary algorithm provided in the brian2modelfitting package (version 0.3). This fitting algorithm (similar to a genetic algorithm) starts with a large set of parameters drawn randomly within set bounds. Based on the fitting performance (i.e. “fitness”; see later discussion on fitting error), some parameter values will have a higher or lower probability of being used in the next iteration. Stochastic perturbations within the parameters allow for an efficient sampling of large parameter spaces^43,44^. The algorithm was initialized with 5000 initial samples of each parameter, and run for 3 iterations. Each parameter was sampled uniformly between upper and lower bounds, based roughly on known biophysical principles (see table S1).

Fitting error was quantified using the root mean squared (RMS Eq. 8) error when the two waveforms (experimentally measured: $\hbox {V}^{\mathrm{e}}$, and model: $\mathrm{V}^{\mathrm{m}}$) were aligned by first spike time (defined as the action potential peak).

8 $$\begin{aligned} \text {RMS} = \sqrt{\frac{\sum \left( V^e-V^m \right) ^2}{n}} \end{aligned}$$

### Experimental methods

To validate the model, brain slices of the pacemaker nucleus were prepared as described previously^10,14^. Briefly, adult black ghost knifefish (*A. albifrons*) were obtained from commercial fish suppliers and housed on a 12/12 light-dark cycle in flow-through tanks at water temperature 27–28 $^\circ$C and conductivity 150–250 $\upmu$ S. All housing and experimental procedures were approved by the Animal Care Committee of the University of Ottawa and were according to the guidelines of the Canadian Council on Animal Care (protocol BL-1773).

Fish (N = 5) were deeply anaesthetized using 0.1% Tricaine methanosulfate (TMS, Syndel International Inc, Nanaimo, BC, Canada) before being transferred to a bath of ice-cold artificial cerebrospinal fluid (ACSF; in mM: 124 NaCl, 24 $\hbox {NaHCO}_{3}$, 10D-Glucose, 1.25 $\hbox {KH}_{2}\hbox {PO}_{4}$ , 2 KCl, 2.5 MgSO$_{4}$ , 2.5 CaCl$_{2}$ ; bubbled with 95% O$_{2}$ /5% CO$_{2}$ ). The brain was quickly removed and the pacemaker nucleus cut away using fine scissors ($\sim \,$1 mm rostral, 2 mm caudal, 1 mm dorsal) and transferred to a 35 mm petri dish perfused with oxygenated room-temperature (22 $^{\circ }$ C) ACSF. After a minimum of 30 min, pacemaker recordings (intracellular and extracellular) were performed with borosilicate glass sharp electrodes (30–90 M$\Omega$, P-2000 electrode puller, Sutter Instrument Company, Novato, CA, USA) using an Axoclamp 2B amplifier (Molecular Devices, Sunnyvale, CA, USA). Data was acquired using a Digidata 1440a digitizer (Molecular Devices) at a sampling frequency of 100 kHz using pClamp 10 (Molecular Devices). Low Na$^+$ ACSF was prepared in a similar fashion as ACSF, only substituting NaCl for equimolar amounts of sucrose (Fisher Chemical, Fair Lawn, NJ, USA). The perfusion system involved a transfer time of approximately 4 min when switching between Na$^+$ and low-Na$^+$ ACSF solutions.

Action potential frequency in the PN was measured from 1-s recordings taken at 20 s intervals using Fourier analysis (as the highest power, dominant frequency). Cessation of spiking was determined when the power at the dominant frequency was less than 1.5 times the power at 60 Hz (signal-to-noise ratio, SNR<1.5). This criterion, along with the dominant frequency being within 5 Hz of a power line harmonic, was additionally used to identify overly noisy recordings prior to cessation; these recordings were removed from the analysis.

## Supplementary information

Supplementary material 1

Supplementary material 2

Supplementary material 3

## Data Availability

All model code and analysis is freely available on GitHub https://github.com/aaronshifman/SCI_REP_2020_Shifman_et_al_Pacemaker_Dynamics
