# Supplementary material for: Dynamics of a neuronal pacemaker in the weakly electric fish Apteronotus
Source: Sci Rep. 2020 Oct 7;10:16707. doi: 10.1038/s41598-020-73566-3 (PMC7542169; doi:10.1038/s41598-020-73566-3)
Supplement: Supplementary file 2 — Supplementary material 2 [file 41598_2020_73566_MOESM2_ESM.pdf]

Dynamics of a neuronal pacemaker in the weakly electric fish *Apteronotus*

Aaron R. Shifman[1,2,3*], **Yiren Sun**[1,2,3], **Chloé M. Benoit**[1,2,3], **and John E. Lewis**[1,2,3]

[1] *Department of Biology, University of Ottawa, Ottawa, Ontario, Canada K1N 6N5*
[2] *Center for Neural Dynamics, University of Ottawa, Ottawa, Ontario, Canada K1N 6N5*
[3] *uOttawa Brain and Mind Research Institute, Ottawa, Ontario, Canada K1H 8M5*
[*] *ashifman@uottawa.ca*

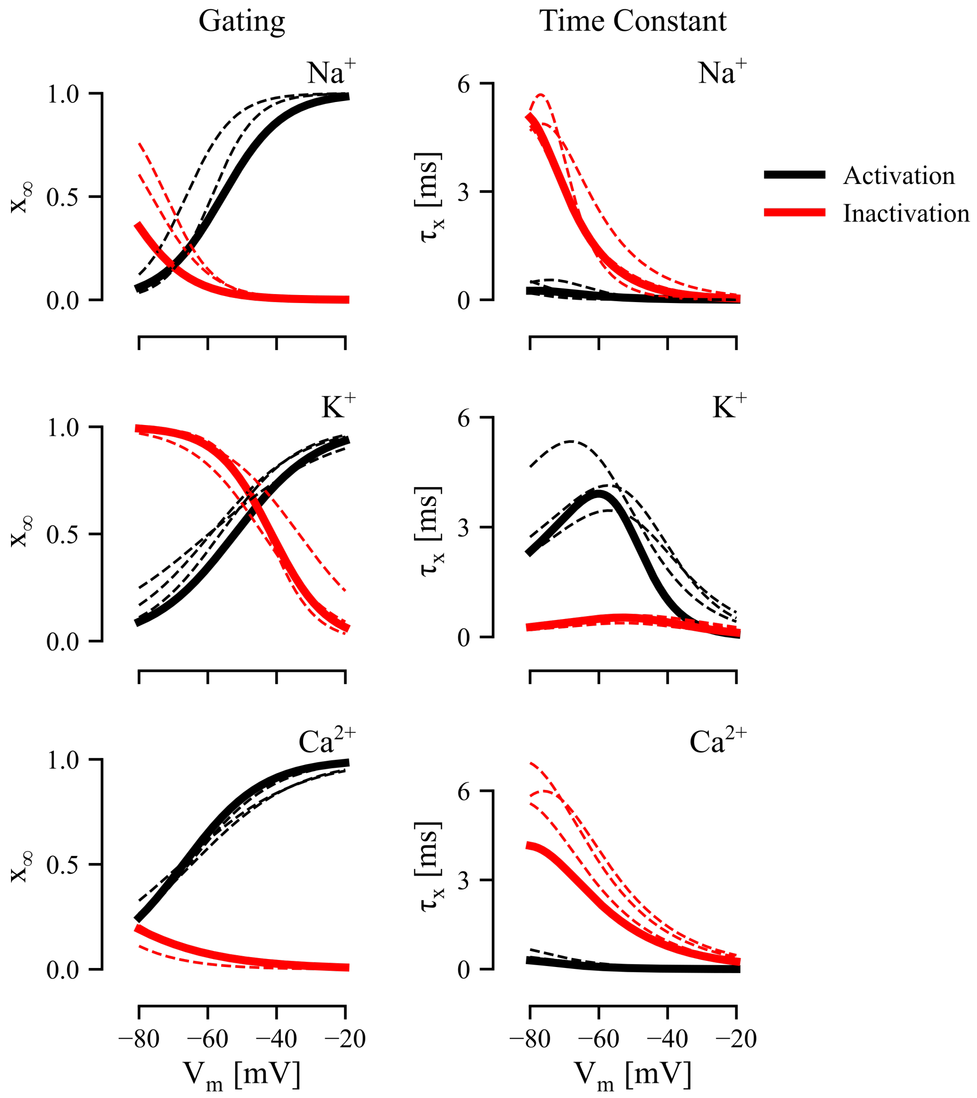

Figure S1. Voltage-dependence functions for each model current across all model fits. Thick lines represent canonical model in figure 1C(i) and dashed lines represent models in figure 1C(ii-iv). Gating functions (left) and time constant functions (right) with rows representing functions for sodium, potassium, and calcium descending.
