# Supplementary material for: Dynamics of a neuronal pacemaker in the weakly electric fish Apteronotus
Source: Sci Rep. 2020 Oct 7;10:16707. doi: 10.1038/s41598-020-73566-3 (PMC7542169; doi:10.1038/s41598-020-73566-3)
Supplement: Supplementary file 3 — Supplementary material 3 [file 41598_2020_73566_MOESM3_ESM.pdf]

Dynamics of a neuronal pacemaker in the weakly electric fish *Apteronotus*

Aaron R. Shifman[1,2,3*], Yiren Sun[1,2,3], Chloé M. Benoit[1,2,3], and John E. Lewis[1,2,3]

[1] *Department of Biology, University of Ottawa, Ottawa, Ontario, Canada K1N 6N5*
[2] *Center for Neural Dynamics, University of Ottawa, Ottawa, Ontario, Canada K1N 6N5*
[3] *uOttawa Brain and Mind Research Institute, Ottawa, Ontario, Canada K1H 8M5*
[*] *ashifman@uottawa.ca*

Parameter	Lower Bound	Upper Bound	Unit
E_{Ca}	20.	30.	mV
E_K	-90.	-80.	mV
E_{Leak}	-90.	-80.	mV
E_{Na}	20.	30.	mV
G_{Ca}	0.	20.	mS
G_K	30.	70.	mS
G_{Leak}	0.	3.	mS
G_{Na}	30.	70.	mS
s_{τ_b}	100.	2.	ms
s_{τ_g}	5.	15.	ms
s_{τ_h}	5.	15.	ms
s_{τ_m}	100.	2.	ms
s_{τ_n}	5.	15.	ms
s_{τ_q}	100.	2.	ms
$\sigma^1_{\tau_b}$	10.	20.	mV
$\sigma^1_{\tau_g}$	10.	20.	mV
$\sigma^1_{\tau_h}$	5.	15.	mV
$\sigma^1_{\tau_m}$	5.	15.	mV
$\sigma^1_{\tau_n}$	5.	15.	mV
$\sigma^1_{\tau_q}$	10.	20.	mV
$\sigma^2_{\tau_b}$	10.	20.	mV
$\sigma^2_{\tau_g}$	10.	20.	mV
$\sigma^2_{\tau_h}$	5.	15.	mV
$\sigma^2_{\tau_m}$	5.	15.	mV
$\sigma^2_{\tau_n}$	25.	35.	mV
$\sigma^2_{\tau_q}$	20.	30.	mV
σ_{b_∞}	10.	20.	mV
σ_{g_∞}	10.	20.	mV
σ_{h_∞}	5.	10.	mV
σ_{m_∞}	5.	10.	mV
σ_{n_∞}	10.	20.	mV
σ_{q_∞}	5.	15.	mV
θ_{b_∞}	-70.	-60.	mV
θ_{g_∞}	-110.	-100.	mV
θ_{h_∞}	-90.	-70.	mV
θ_{m_∞}	-70.	-50.	mV
θ_{n_∞}	-65.	-45.	mV
θ_{q_∞}	-55.	-25.	mV
θ_{τ_b}	-100.	-80.	mV
θ_{τ_g}	-85.	-75.	mV
θ_{τ_h}	-90.	-60.	mV
θ_{τ_m}	-90.	-70.	mV
θ_{τ_n}	-65.	-45.	mV
θ_{τ_q}	-55.	-35.	mV

Table S1. Upper and lower bounds for parameter selection in the GA

Parameter	**Canonical Fit**	Model ii Fit	Model iii Fit	Model iv Fit	Unit
E_{Ca}	23.95	22.13	29.01	27.02	mV
E_K	-80.87	-87.12	-84.49	-89.02	mV
E_{Leak}	-88.91	-84.63	-88.95	-87.81	mV
E_{Na}	24.22	25.56	22.12	21.06	mV
G_{Ca}	14.28	4.13	1.99	2.57	mS
G_K	59.27	50.16	39.90	33.16	mS
G_{Leak}	1.13	1.98	1.11	2.17	mS
G_{Na}	63.13	52.48	48.66	61.82	mS

Table S2. Ionic Parameters for model fits. Canonical Model maps to Figure 1A, Model B-D maps to Figure 1B-D

Parameter	**Canonical Fit**	Model ii Fit	Model iii Fit	Model iv Fit	Unit
s_{τ_b}	0.62	1.38	1.65	1.07	ms
s_{τ_g}	8.28	11.36	11.95	14.02	ms
s_{τ_h}	10.29	11.36	9.71	9.62	ms
s_{τ_m}	0.50	0.47	1.08	1.33	ms
s_{τ_n}	6.56	9.69	7.18	6.35	ms
s_{τ_q}	1.01	0.72	1.15	0.96	ms
$\sigma^1_{\tau_b}$	11.27	11.31	13.50	18.50	mV
$\sigma^1_{\tau_g}$	17.94	17.33	17.63	17.60	mV
$\sigma^1_{\tau_h}$	11.15	7.27	13.49	13.01	mV
$\sigma^1_{\tau_m}$	11.98	7.20	8.86	8.94	mV
$\sigma^1_{\tau_n}$	7.17	12.68	10.72	13.23	mV
$\sigma^1_{\tau_q}$	13.14	13.41	17.87	17.79	mV
$\sigma^2_{\tau_b}$	12.62	15.89	17.79	18.41	mV
$\sigma^2_{\tau_g}$	14.99	17.95	15.38	17.56	mV
$\sigma^2_{\tau_h}$	10.26	7.80	11.14	8.17	mV
$\sigma^2_{\tau_m}$	13.52	7.70	12.87	14.10	mV
$\sigma^2_{\tau_n}$	26.62	32.07	33.81	31.13	mV
$\sigma^2_{\tau_q}$	25.15	25.97	28.51	22.07	mV
σ_{b_∞}	11.55	15.12	16.80	12.37	mV
σ_{g_∞}	18.38	12.71	16.72	18.55	mV
σ_{h_∞}	9.48	9.03	8.51	6.92	mV
σ_{m_∞}	8.78	6.91	6.33	9.08	mV
σ_{n_∞}	12.05	12.99	11.33	18.22	mV
σ_{q_∞}	8.03	6.71	11.40	10.39	mV
θ_{b_∞}	-67.10	-64.67	-67.86	-65.61	mV
θ_{g_∞}	-106.52	-106.48	-102.24	-106.40	mV
θ_{h_∞}	-85.67	-84.66	-76.30	-72.08	mV
θ_{m_∞}	-55.85	-66.36	-58.86	-55.27	mV
θ_{n_∞}	-52.16	-59.15	-56.39	-59.78	mV
θ_{q_∞}	-41.48	-42.43	-33.52	-43.99	mV
θ_{τ_b}	-83.44	-96.35	-88.60	-94.56	mV
θ_{τ_g}	-82.37	-83.12	-77.18	-82.55	mV
θ_{τ_h}	-82.53	-76.68	-77.66	-84.61	mV
θ_{τ_m}	-77.87	-85.17	-72.28	-85.84	mV
θ_{τ_n}	-52.65	-59.64	-47.93	-49.18	mV
θ_{τ_q}	-47.45	-46.91	-44.41	-45.09	mV

Table S3. Gating Parameters for model fits. Canonical Model maps to Figure 1A, Model B-D maps to Figure 1B-D
